# Clinic Atlanta Medical College

**Published:** 1875-04

**Authors:** L. K. Philpot, A. W. Calhoun

**Affiliations:** Reporter; Professor of Diseases of the Eye and Ear


### Clinic Atlanta Medical College.

L. K. PHILPOT, Reporter.

A. W. CALHOUN., M.D., Professor of Diseases of the Eye and Ear.

(*Continued from page* 605 *January Journal.*)

Case 10. *Exanthematous Ophthalmia.*—E. M., col'd, æt. three years, Jonesboro, Ga. In this case we have a combination of diseases. Upon the face and over part of the scalp you will observe the disease known as acute eczema, and from the skin the inflammation has extended into the eye, upon the mucous lining of the lids and ball, producing the above named affection. There is an eruption upon the cornea, (Phlyctenulæ) particularly at its sclerotic margin, which, with the general appearance of the child, indicates a scrofulous taint of the system. As the disease of the eye appeared after that of the face, it is probable that the eczema is the immediate exciting cause of the scrofulous trouble upon the cornea, and when this (the eczema) is relieved, the eye trouble will of itself pass away. But in consequence of the cornea being diseased, this must be treated, lest the disease extend still deeper. A solution of atropine (gr. ij. to ℥j.) will be applied several times daily, by dropping a few drops upon the conjunctiva of the lower lid. As in the other cases you have seen, this will dilate the pupil and have a tendency to relieve the sensitiveness of the cornea and overcome the photophobia. For the eczema upon the face and scalp, we will prescribe the following:

℞.—Ung. Diacyli (Hebræ), . . . . . ℥ij.

Ol. Lavendulæ, . . . . . . . gtt. v.

M. and Ft. Ung.—S. Spread upon strips of cloth and apply to the diseased surface, binding it by means of a bandage or a cloth cap made for the purpose. To be changed twice daily. Where the hair upon the scalp interferes materially with the application of the ointment, it must be cut quite short, or even shaved away.

Note.—With this treatment alone, both the eczema and the conjunctivitis rapidly subsided, and the case was dismissed as cured, after two weeks.

Case 11. *Scrofulous Ophthalmia, with a small Corneal Abscess.*—A. M., col'd girl, æt. nine years, Atlanta. This is a specially interesting case, on account of the existence of a small abscess imbeded between the lamellæ of the cornea. In addition to the ordinary symptoms of scrofulous inflammation of the eye, you find in the cornea, just below the pupil, a small circumscribed spot, bulged a little forward and presenting a yellow appearance, somewhat like a drop of pus when seen under a thin glass. In discussing this subject, you will remember that it was recommended to freely open the corneal abscess, from whatever cause produced, whenever it was found to be on the increase and threatening a still further destruction of the tissues. In this case it is circumscribed, and from the history of the disease and the present appearance of the abscess, we judge it to be stationary and not demanding the use of the knife. Should it, however, extend, this treatment must be resorted to. For the present we will use a solution of atropine freely and often, for reasons which you have several times had explained to you. Also the compound belladonna ointment, which consists of—

| | |
|---|---|
| ℞.—Ext. Belladonnæ, . . . . . | gr. xvj. |
| Hyd. Præcip. Albi, . . . . . | gr. xij. |
| Ung. Aquæ Rosæ (or Ung. Simp.), . . | ʒij. |

M. and Ft. Ung.—Rub a small piece over the forehead, above the eye, every two or three hours; and for its alterative effect we prescribe the following:

| | |
|---|---|
| ℞.—Fl. Ext. Stillingiæ. | |
| Tinct. Gentianæ, . . . . . | aa. ℥jss. |
| Iod. Potass., . . . . . . | ℥ss. |
| Hyd. Corros. Sublim., . . . . | gr. j. |
| Aquæ Destil, . . . . . . | ℥iij. |

S.—One teaspoonful three times daily.

The eye is to be kept bandaged, and thus excluded from the light and allowed complete rest. If necessary, warm applications will be made over the eye. Patient will also take an occasional dose of saccharated calomel, and live upon a nourishing diet and take plenty of outdoor exercise.

NOTE.—After several months' treatment the patient has fully recovered, and her general health is thoroughly restored. A small opacity occupies the seat of the abscess, but it is slowly disappearing, and will probably pass away altogether.

CASE 12. *Keloid Tumor upon the lobe of each Ear—Extirpation.* A. H., col'd girl, æt. eleven years, Atlanta. Hanging from the lobe of the left ear, and embracing the lobe itself, is a tumor of the size of an English walnut. These keloid tumors mostly spring from an old scar, and sometimes grow to be quite large. Amongst negroes you will find them most frequently, and the lobe of the ear is a favorite seat for them. As in this case, they are often produced by wearing brass ear-rings, the brass irritating and poisoning the tissues in such a way as to produce these peculiar growths. As a rule they are not painful unless pressed upon. The only treatment is their removal by means of the knife. The tumor is excised in such a way as to leave the lobe in as good a shape as possible, and the edges of the wound brought together with silk sutures. Wound healed rapidly, and only by comparing the two ears can it be seen that any part of the left lobe has been removed. Two weeks after the first operation, a smaller tumor of the same character, (keloid,) upon the lobe of the right ear, and produced by the same cause (wearing brass ear-rings) was removed just as in the first instance, and with like good result. Keloid growths are prone to return, even after the most complete removal, and in dismissing the patient as cured, she was directed, in case of their return, to have them excised before they grew to any size, as this would probably check their reappearance.

CASE 13. *Exanthematous Ophthalmia.*—S. W., White, æt. two years, Atlanta. This is a typical case of exanthematous conjunctivitis, being free from any complications, as existed in the similar case which was brought before you. The conjunctivitis is nothing more than an inflammation produced by the presence of an acute eczema upon the face and lids. The cure of the affection of the conjunctiva requires only the removal of the disease which caused it. For the eye we will recommend nothing more than the frequent cleansing from the secretions which may collect underneath the lids, and the application of simple cold water.

For the eczema the diacylon ointment (ung. diacyli (Hebræ) ℥ij., ol. lavendulæ, gtt. v.) is to be used in the same manner as in the other case.

Note.—One week later you find the skin disease materially diminished, and the conjunctivitis quite gone. Treatment to be continued until the original trouble (the eczema) is cured.

Case 14. *Central Opacity of the Cornea*—Iridotomy.—Wm. B., white, æt. twenty-five years, Campbellton, Ga. The patient has had granular lids in both eyes, producing an ulcer upon the cornea of the left. By long treatment with nit. silver, sulph. copper, etc., the granulations have disappeared, and the ulcer has healed, but left in its stead a thick opacity, situated directly in front of the pupil and interfering with vision. Upon dilating the pupil and examining the interior of the eye with the ophthalmoscope, every thing within was found to be perfectly normal; and with the pupil in this dilated condition, the vision (particularly for distant objects) was immensely improved, the clear portion of the cornea around the opacity permitting the free entrance of the rays of light into the interior. Formerly an *iridectomy* was the remedy against such an evil; but this has many disadvantages connected with it. An iridotomy answers the same purpose as an iridectomy in such cases, (viz., the making of an artificial pupil,) and yet avoids the disadvantage of a portion of the iris being cut away. Here we intend making the first of these operations. The patient is placed upon the table, the lids held apart by means of the eye speculum, and grasping with a pair of forceps a fold of conjunctiva below the cornea, the spear-shaped iridectomy knife is passed into the anterior chamber, through the upper part of the corneo-sclerotic junction. The closed blades of De Wecker's scissors are then entered through this opening into the anterior chamber, until the point is opposite the lower pupillary margin, when the blades are allowed to open, and one passed through the pupil behind the iris and resting upon the anterior capsule of the lens. The other passes in front of the iris, and the instrument is pushed on towards the bottom of the anterior chamber, until a sufficient portion of the iris is brought between the two blades. These are then brought together and the iris tissue severed, and by the contraction of the divided circular muscular fibres (sphincter), a triangular opening is made, the base looking towards the pupillary border. The prolapsed portion of iris is replaced and the eye bandaged.

When seen on the following day, and after the anterior chamber had refilled, the cut edges of the iris had drawn still further apart, and a sufficiently large artificial pupil existed below the corneal opacity to permit of good vision, and no portion of the iris had been cut away, as would have been had an *iridectomy* been made. Patient soon recovered, with a good (artificial) pupil and greatly increased vision. No anæsthetic was used in this case.

CASE 15. *Staphyloma—Operation, and tattooing the Corneal Opacity.*—M. B., col'd woman, æt. thirty years, Atlanta. From some severe inflammation, the patient has had the cornea of the right eye injured to such an extent that a partial staphyloma has been the result. She has no vision in the eye, and an operation is necessary only to reduce the size of the protrusion, when whatever opacity may be remaining after the operation has healed will be changed from its white to a dark appearance, by the process of tattooing.

A Beer's knife is passed through the lower portion of the staphyloma, and, with forceps and scissors, a good-sized piece of the protruding cornea is cut away. The eye is bandaged and the wound allowed to heal by granulation, which it does in the course of three or four weeks, the contraction of the cicatrix reducing the projection to its natural size, and leaving a flat, white opacity. To relieve this deformity it is proposed to darken the opacity by tattooing with Indian ink. An eye speculum is placed between the lids, and, with the patient in a recumbent position, a semi-fluid solution of the Indian ink is spread over the opacity, and with the tattooing instrument (eight or ten needles fastened in a handle) a multitude of little holes are rapidly pricked into the parts, into which the ink flows and remains, becoming part and parcel of the tissue. The ink and the needles are several times reapplied during the one operation, doing as much as it is prudent for one time. After an interval of a week the operation is again and again repeated in the same way, until the formerly white appearance of the front part of the eye has given way to a dark color. Very little irritation has followed each application, the eye recovering fully after a few days. The conspicuous, white appearance has been removed, and only when tolerably near the patient can the nature of the formerly existing trouble and the operation be observed.

Tattooing is also applicable to small corneal opacities, rendering white spots colored, and hence less conspicuous.

CASE 16. *Hordeolum, Treatment to prevent Styes.*—T. E. J., white,

æt. twenty-one years, Georgia. This case is presented to you simply to show you, anatomically, what a stye is and its treatment, and to speak of the mode best calculated to prevent its return. Inflammation and swelling of the tissues in and around the hair follicles and in the little glands which supply them, constitute a stye. It points, at the margin of the lid, or near it, upon the conjunctival surface. The quickest way of relieving it is by puncturing and letting out whatever pus may be contained within, or even though pus may not yet have formed, the bleeding after puncturing often brings about a speedy cure. At the same time, use warm poultices. Many persons (particularly delicate individuals who live high) seem to be peculiarly susceptible to this annoying disease, and have frequent returns of it without any apparent cause, or upon the slightest exposure. To get thoroughly rid of this tendency to recurrence, not unfrequently requires no small amount of patience and perseverance on the part both of the physician and the patient. I have found an ointment composed of gr. ij.–iv. nit. silver to ℥j. simple cerate, rubbed well into the edges of the lids once or twice daily, to be beneficial in preventing relapses. Also the ointment of red oxide mercury, gr. ij.–iv. to ʒj. cerate, used in the same way, effects a perfect cure if persistently kept up. It must be rubbed into the edges of the lids every night on going to bed, and left on during the entire night. If the health of the patient requires it, give also constitutional remedies. This mode of treatment will rarely fail to check the recurrence of the disease.

Case 17. *Scrofulous Ophthalmia with Pannus.*—E. McC., col'd, æt. twenty-one years, Atlanta. Similar cases to this you have seen on several previous occasions, but I would like to call your attention again to the frequency of the existence of pannus as a result of scrofulous ophthalmia. You have now had frequent opportunity of seeing it, following this disease and also granular lids, and, in the majority of instances, you will find it to be the result of the one or the other. Treating the original trouble, by means of atropine, compound belladonna ointment, and, if necessary, the yellow oxide of mercury ointment, with constitutional remedies, will overcome it, and the pannus will of itself get well. In your practice you will notice that negroes are peculiarly liable to scrofulous diseases, manifesting itself most frequently in the eye. Their susceptibility, no doubt, depends upon their irregular mode of living, want of care and attention to themselves. Consumption is yearly carrying off thousands of them.

